# Mass spectrometry-based approaches to targeted quantitative proteomics in cardiovascular disease

**DOI:** 10.1186/s12014-016-9121-1

**Published:** 2016-10-05

**Authors:** Clementina Mesaros, Ian A. Blair

**Affiliations:** 1 Penn SRP Center and Center of Excellence in Environmental Toxicology, Department of Systems Pharmacology and Translational Therapeutics, University of Pennsylvania, Philadelphia, PA 19104 USA; 2 BluePen Biomarkers, 3401 Grays Ferry Avenue, Philadelphia, PA 19146-2799 USA

**Keywords:** Apolipoprotein AI, Apolipoprotein CIII, C-reactive protein, COX-2, Multiple reaction monitoring, Stable isotopes, Electrospray ionization, UPLC

## Abstract

Mass spectrometry-based proteomics methodology has become an important tool in elucidating some of the underlying mechanisms involved in cardiovascular disease. The present review provides details on selected important protein targets where highly selective and specific mass spectrometry-based approaches have led to important new findings and provided new mechanistic information. The role of six proteins involved in the etiology of cardiovascular disease (acetylated platelet cyclooxygenase-1, serum apolipoprotein A1, apolipoprotein C-III, serum C-reactive protein, serum high mobility group box-1 protein, insulin-like growth factor I) and their quantification has been discussed. There are an increasing number of examples where highly selective mass spectrometry-based quantification has provided new important data that could not be obtained with less labor intensive and cheaper immunoassay-based procedures. It is anticipated that these findings will lead to significant advances in a number of important issues related to the role of specific proteins in cardiovascular disease. The availability of a new generation of high-resolution high-sensitivity mass spectrometers will greatly facilitate these studies so that in the future it will be possible to analyze serum proteins of relevance to cardiovascular disease with levels of specificity and/or sensitivity that cannot be attained by immunoassay-based procedures.

## Introduction to targeted proteomics and cardiovascular disease

**Fig. 1:**
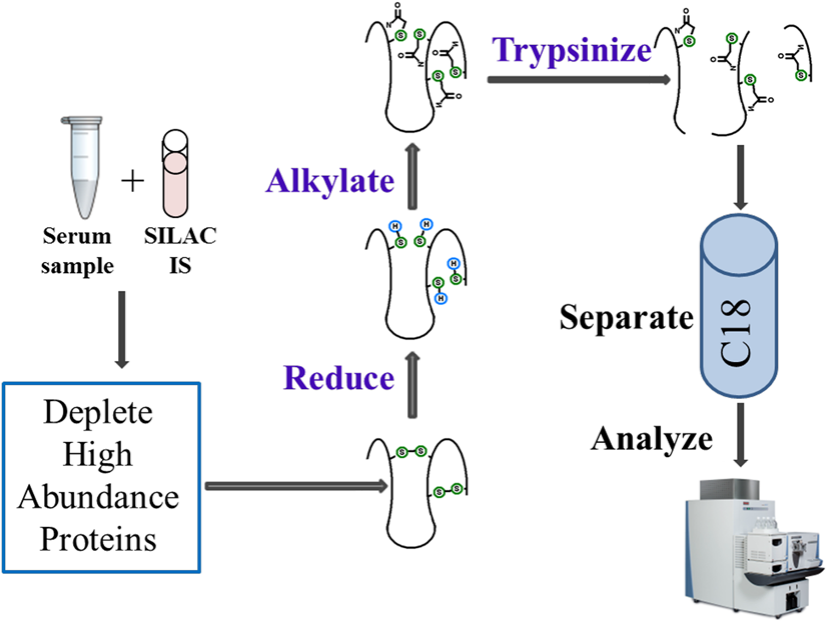
Workflow for targeted proteomics

Proteomics is increasingly providing clues to the underlying mechanism of cardiovascular disease [1]. However in order to translate these more global findings it is often necessary to take a more targeted mass spectrometry (MS)-based approach, particularly when dealing with patient-derived biofluids [2]. The use of liquid chromatography-multiple reaction monitoring (LC–MRM/MS) or LC-high resolution (HR) MS for quantification of selected proteins has several key advantages when compared with antibody-based methods. The general workflow for targeted proteomics (Fig. 1) starts by adding a stable isotope labeled protein internal standard, such as a stable isotope labeling by amino acids in cell culture (SILAC) standard, to a serum or plasma sample. This standard has identical physicochemical properties to the endogenous protein and so it corrects for any losses that occur during the analytical procedure as well as acting a carrier for low abundance proteins. The serum or plasma sample is then mixed with buffer and the sample is depleted of highly abundance proteins by a variety of techniques such as antibody columns, polyacrylamide gel electrophoresis, or immunoprecipitation (Fig. 1). Proteins are dissolved in urea or thiourea with ammonium bicarbonate, reduced with dithiothreitol, alkylated with iodoacetamide, and digested with a protease such as trypsin. The resulting digest is desalted and analyzed by reversed-phase LC coupled to a triple quadrupole or HR mass spectrometer (Fig. 1). Analyte detection by LC–MRM/MS is essentially structurally unambiguous by offering absolute selectivity. Even in instances where selectivity may be compromised by a co-eluting analyte, with isobaric precursor and product ions, these interferences can often be identified and the method altered to eliminate the problem. The ability to assay multiple peptides derived from multiple proteins in a single LC–MRM/MS analysis permits multiplexing to be readily conducted. These factors, coupled with the use of stable isotope labeled protein internal standards, offer a level of specificity and flexibility that cannot be attained by standard immunoassay-based methodology. The present review will focus on selected important protein targets where a highly selective and specific approach has led to important new findings and provided new mechanistic information. The quantification of six proteins (Table 1), acetylated platelet cyclooxygenase-1 (platelet Ac-COX-1) [3], serum apolipoprotein A1 (ApoA-I) [4], serum ApoC-III [5], serum C-reactive protein (CRP), serum high mobility group box-1 (HMGB1) [6], and serum insulin-like growth factor I (IGF-I) [7, 8], which are involved in various aspects of cardiovascular disease will be discussed in detail below. Ultimately, it could be that these quantitative methods will simply serve as gold standards for other less specific technologies. Therefore, in the future, it is conceivable that less labor intensive and less expensive approaches can be developed.

**Table 1:** Summary of assays used for measurements of proteins biomarkers of cardiovascular disease

Protein	LC–MS assay	References	Immunoassay	References
Ac-COX-1	SID LC–MRM/MS	[13]	None available	
ApoA-I	SID LC–MRM/MS	[4]	ELISA	[24]
ApoC-III	LC–HRMS	[5]	ELISA	[40–43]
hsCRP	LC–MRM/MS	[62–67]	ELISA	[54–60]
HMGB1	LC–MRM/MS	[79, 82, 88]	ELISA	[83–87]
IGF-I	LC–HRMS	[108]	ELISA	[104–107]

### Platelet Ac-COX-1

COX-1 is a prostaglandin synthetase that is responsible for the production of proinflammatory prostaglandins (PGs) from arachidonic acid, a 20 carbon fatty acid, at a site of injury. Aspirin, which was first synthesized in 1893, is a non-selective irreversible COX inhibitor. It is one of the world’s most widely used therapeutic agents through its effectiveness in reducing pain, fever, and inflammation. Acetylation of platelet COX-1 to form Ac-COX-1 occurs after aspirin treatment in vivo through acetylation of serine-529 in the hydrophobic substrate binding channel [9]. This irreversibly inactivates the platelet COX-1 enzyme. Aspirin is classified as a non-steroidal anti-inflammatory drug (NSAID) but it differs from traditional NSAIDs (tNSAIDs), which inhibit platelet COX-1 through reversible non-covalent interactions. These tNSAIDs prevent the binding of aspirin to serine-529 by occupying the substrate binding channel of platelet COX-1 [9]. In 2010, some 12.8 % of US adults (29.4 million) were estimated to be taking tNSAIDs regularly and 19.0 % (43.6 million) were estimated to be taking aspirin chronically for cardioprevention [10]. Consequently, chance alone suggests that approximately 2.4 % of US adults (5.5 million) were chronically exposed to both a NSAID and aspirin in 2010. Therefore, it is necessary to assess the antiplatelet effects of tNSAIDs (due to reversible COX-1 inhibition), the inhibition of aspirin binding to platelet COX-1 by particular tNSAIDs, and the irreversible antiplatelet effects of aspirin (due to irreversible COX- inhibition) during co-administration of particular tNSAIDs. This is extremely challenging using conventional methodology [11] but particularly important for assessing the effects of tNSAIDs with variable and extended half-lives such as naproxen [12].

In order to address this complex problem, a method for the direct quantification of Ac-COX-1 was developed using a proteomics approach in combination with stable isotope dilution liquid chromatography-multiple reaction monitoring (LC–MRM)/MS [13]. This has made it possible to analyze the ability of structurally distinct tNSAIDs to inhibit Ac-COX-1 formation through drug–drug interactions in vivo. Initially, combinations of proteases were screened in order to find the best way to efficiently excise a peptide containing the acetylated serine-529 from aspirin-modified COX-1. It was found that a combination of two proteases, Glu-C—a serine protease that cleaves at the C-terminal side of aspartic or glutamic acid residues and trypsin - a serine proteases that cleaves primarily at the C-terminal side of lysine or arginine, except when either is followed by proline, efficiently hydrolyzed COX-1 to yield the octapeptide –I^524^GAPFSLK^531^, which had excellent MS ionization characteristics as both the acetylated and non-acetylated forms. The stable isotope dilution LC–MRM/MS assay that was developed provided a highly precise and accurate way to assess the amount of platelet Ac-COX-1 that was formed in vivo during the administration of various NSAID combinations [3]. Aspirin was shown to acetylate primarily the catalytic monomer (rather than the allosteric monomer) within the homodimeric COX-1 complex [14]. The stable isotope dilution LC–MRM/MS assay revealed that maximum acetylation occurred on >50 % of the COX-1 molecules [3]. Therefore, either a fraction of Ac-COX-1 monomers was not assembled into homodimers and/or both the catalytic and allosteric monomers were acetylated in some of the homodimers.

The study by Li et al. [3] simulated the chronic dosing pharmacokinetics of ibuprofen, naproxen, and celecoxib and compared the expected steady-state plasma concentrations with those observed 2 h after single dose. Chronic dosing with ibuprofen and naproxen in the dose range used in the PRECISION trial (Prospective Randomized Evaluation of Celecoxib Integrated Safety Versus Ibuprofen or Naproxen) and the Standard Care Versus Celecoxib Outcome Trial would result in plasma concentrations that are similar to or higher than the concentrations that were found to prevent COX-1 acetylation. This means that it is likely that platelet inhibition by aspirin will be blocked in patients on these dosing regimens, irrespective of the order in which aspirin and the NSAID are taken. The levels simulated with celecoxib are in the range of the plasma concentrations that were observed to have little or no effect on aspirin acetylation.

Fasted healthy volunteers received a single dose of aspirin (325 mg) to assess their aspirin-responsiveness. In the second study period, after a washout period of at least 2 weeks, subjects received a single dose of 600 mg ibuprofen, 500 mg naproxen or 200 mg celecoxib in a sequential treatment group with an open-label design. Two hours after the NSAID, a single dose of aspirin (325 mg) was administered. Platelet Ac-COX-1, arachidonic acid-induced platelet aggregation, platelet TxB2, and urinary 11-dehydro TxB2 were also measured before NSAID administration, before aspirin administration, and 24 h thereafter. As noted above, the octapeptide (I^524^GAPFSLK^531^) containing the acetylation site, serine-529, from COX-1 was used for quantification. Proteins isolated from washed platelets were separated on a gel and proteolyzed in the presence of the stable isotope-labeled internal standards. Assay precision and accuracy was excellent with limits of detection and quantification being 200 amol and 3 fmol, respectively.

The LC–MRM/MS assay revealed that there were potent drug–drug interactions between ibuprofen and aspirin and between naproxen and aspirin [3]. Furthermore, a reduction of platelet Ac-COX-1 by just slightly more than 10 % resulted in the loss of aspirin-mediated inhibition of platelet function. This latter finding is consistent with the large functional COX-1 reserve available in platelets [15]. Overall, these findings illustrate how difficult it will be to make head-to-head comparisons of structurally distinct NSAIDs in clinical studies designed to assess their cardiovascular risk when aspirin consumption has not been addressed.

### ApoA-I

ApoA-I is the major protein constituent of high density lipoprotein (HDL) particles, accounting for approximately 65 % of the protein mass [16]. It is a 45.4 kDa protein that is composed of 396 amino acids. Its biosynthesis occurs primarily in the liver and small intestine as a pre-pro-protein. This protein is cleaved and secreted from the liver and intestine as pro-ApoA-I into the blood circulation or the lymphatic system. After cleavage of the pro-section, the resulting mature ApoA-I is converted into lipid-poor ApoA-I particles that absorb free cholesterol. A number of metabolic steps then take place including the action of lecithin-cholesterol acyltransferase (LCAT) in the particles, which rapidly esterifies cholesterol into cholesteryl esters (CEs) to generated larger HDL particles. In addition, cholesteryl ester transfer protein (CETP) facilitates the exchange of CE for triacylglycerols (TAGs) between ApoB-containing lipoproteins and HDL particles. After a series of complex metabolic steps [16], the mature HDL particles bind to scavenger receptor class B type 1 (SR-B1) or to holo-HDL receptors on the liver where TAGs and cholesterol esters are selectively absorbed into the liver [17]. This removes cholesterol from cells and the circulation to the liver, a process that is known as reverse cholesterol transport [18]. The ability of HDL to shuttle cholesterol and TAGs from the circulation into the liver provides the most likely explanation for the inverse relationship between serum HDL concentrations and risk for coronary heart disease (CHD) risk [19, 20]. It has been estimated that a 1 % increase in serum HDL is associated with a 2 % reduction in the risk to develop CHD [21].

The critical role of endogenous ApoA-I biosynthesis in the assembly of HDL particles has stimulated numerous pharmacological approaches to elevating serum ApoA-I levels [17]. Statins, which are HMG-CoA reductase inhibitors, have proved to be the most successful class of drug for increasing HDL levels and lowering CHD risk [22]. This reduction in risk occurs through increasing HDL concentrations as well as by lowering low density lipoprotein (LDL) concentrations [17]. The lower LDL concentrations occur through inhibition of HMG-CoA reductase and inhibition of cholesterol biosynthesis [23]. HDL concentrations are thought to be increased through a reduction of geranylgeranyl pyrophosphate biosynthesis, which in turn prevents geranylgeranylation of RhoA leading to an increase in PPARα—a transcription factor for ApoA-I [23].

**Fig. 2:**
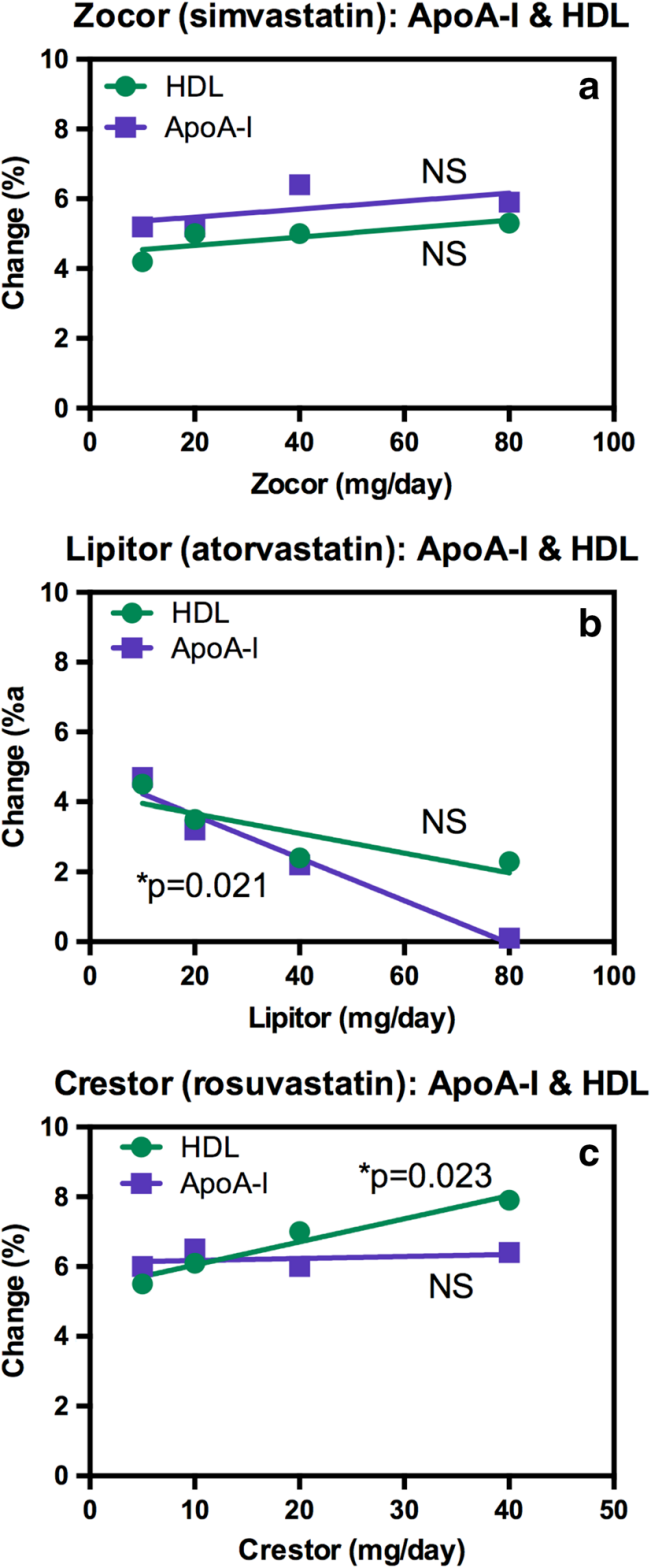
Percent change from baseline in serum HDL and ApoA-I in 32,258 patients from 37 randomized studies. Patients were treated with different doses of statins. **a** Zocor (simvastatin). **b** Lipitor (atorvastatin). **c** Crestor (rosuvastatin). Redrawn with permission from [24]. *NS* not significant

Although, there is clear evidence that up-regulation of PPARα by statins results in increased ApoA-I, there is a curious discrepancy on the reported efficacy of different statins [24]. In a retrospective analysis of data from 37 trials involving 32,258 patients, there was no increase in HDL levels from increasing doses of Zocor (simvastatin, Fig. 2a) and Lipitor (atorvastatin, Fig. 2b) and no increase in ApoA-I levels for Zocor (Fig. 2a). Paradoxically, Apo-A-I levels were reported to actually decline with increasing doses of Lipitor even though HDL levels remained constant (Fig. 2b). The most potent of the statins (Crestor, rosuvastatin) actually caused a modest increase in HDL levels with increasing concentrations of the drug but there was no concomitant increase in serum ApoA-I concentrations (Fig. 2c). The lack of correlation between HDL concentrations and serum ApoA-I suggests that perhaps there could be a problem of specificity with the enzyme-linked immunosorbent assay (ELISA) used to analyze serum ApoA-I. It is conceivable that the three statins could have differential off-target anti-inflammatory effects at higher doses. However, it is difficult to reconcile such potential off-target effects with decreased serum ApoA-I levels such as those observed with Lipitor (Fig. 2b).

**Fig. 3:**
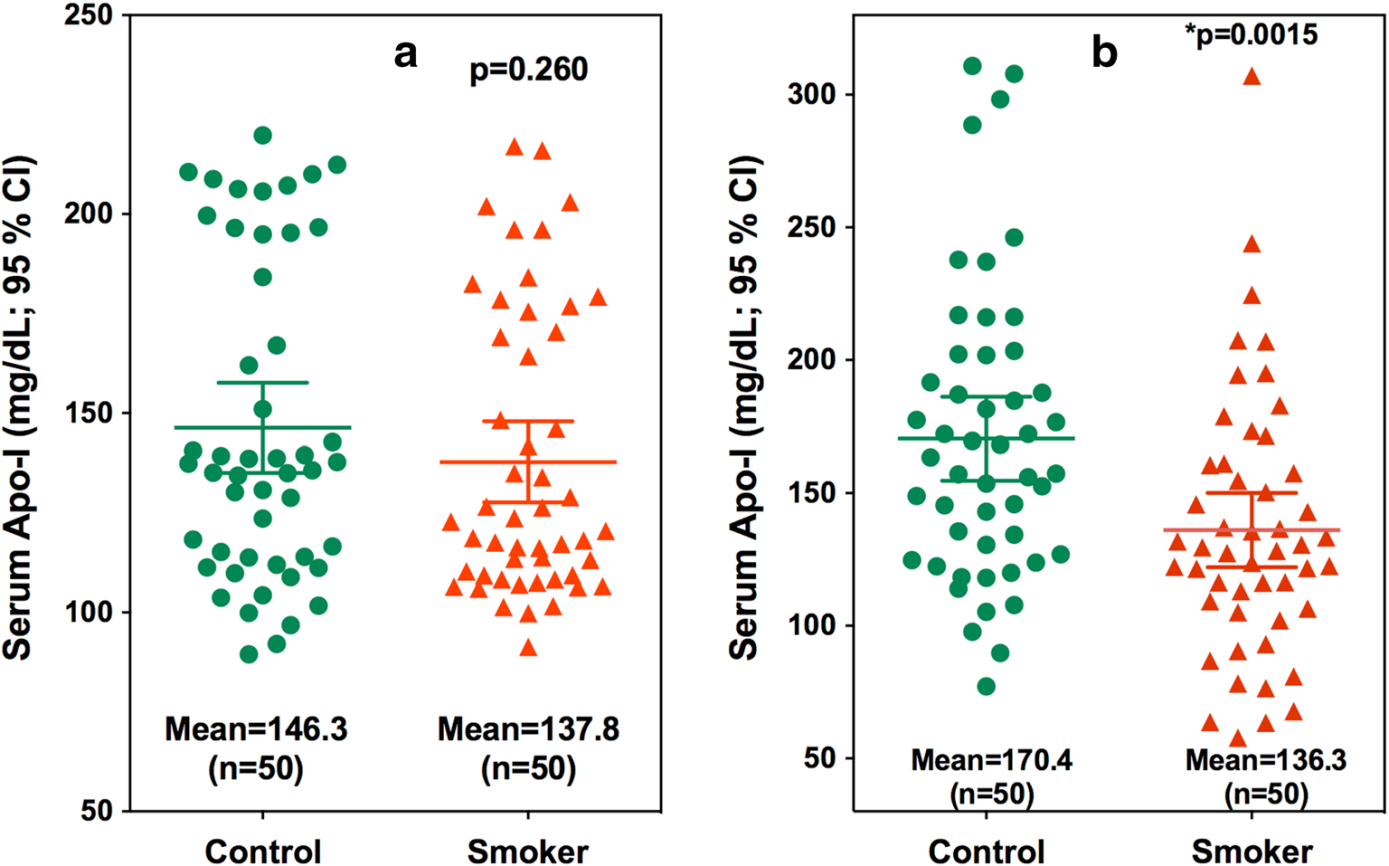
Serum ApoA-I levels in control non-smokers and tobacco smokers. **a** Analysis by ELISA. **b** Analysis by stable isotope dilution LC–MRM/MS using a SILAC labeled Apo-A-I internal standard. Re-drawn with permission from [4]

In keeping with the possible lack of specificity for serum ApoA-I, no ELISA-based studies have detected decreased serum ApoA-I levels in samples obtained from cigarette smokers in spite of their reduced HDL levels when compared with non-smokers [25, 26] and their approximately 30 % increase in risk for CHD [27]. Our group addressed this potential problem recently. Using a conventional ELISA, we were unable to detect any differences in serum ApoA-I levels between smokers and non-smokers (Fig. 3b) [4]. However, a more specific stable isotope dilution LC–MRM/MS assay revealed that there was in fact an almost 20 % decrease in serum ApoA-I levels in smokers when compared with nonsmokers (Fig. 3b) [4]. This finding is consistent with a significantly increased risk for CHD among smokers [27]. In light of our data, previous studies on ApoA-I levels that were determined by ELISA methodology such as the studies on statins discussed above [24] will have to be re-evaluated using more specific stable isotope dilution LC–MRM/MS-based methodology.

Quantitative immunoassays such as ELISAs are widely used for quantifying protein biomarkers due to their high-throughput capabilities and high sensitivity [28]. Immunoassays are based on epitope recognition and can suffer from technical problems including weak antibody affinity, high cross-reactivity and lack of concordance among platforms [29]. Stable isotope dilution LC-selected reaction monitoring (SRM)/MS or LC–MRM/MS can provide a solution to these potential problems [4]. Quantification of ApoA-I by LC–SRM/MS or LC–MRM/MS employing Absolute Quantification (AQUA) peptides has been reported for purified ApoA-I, in serum and plasma [4]. Although AQUA peptides were added before the tryptic digestion, formation and decomposition of individual monitored peptides was highly variable. This variability suggests that the selection of rapidly formed peptides with no or minor missed cleavages and the use of short trypsin incubation times were likely to reduce the accuracy. AQUA approach is recommended for use with proteins that readily undergo digestion to peptides that are stable to further hydrolysis.

Stable isotope labeling by amino acids in cell culture (SILAC)-based strategies can provide the most accurate and precise methods for absolute protein quantification. The use of SILAC-labeled internal standards minimizes differences in sample processing and proteolytic digestion between the standard and its endogenous counterpart. We developed a method using a spike-in SILAC approach to quantify ApoA-I in human serum [4]. The SILAC-labeled ApoA-I internal standard used was labeled with [^13^C_6_^15^N_2_]-lysine and [^13^C_9_^15^N_1_]-tyrosine and expressed in the human kidney HEK293 cell line. The recombinant labeled ApoA-I was expressed without a tag. SILAC-labeled ApoA-I was spiked into serum samples at the beginning of the sample preparation procedure—before electrophoresis separation and tryptic digestion. We compared the measured ApoA1 in 50 smokers and 50 nonsmokers together with the ones from a commercial ELISA kit.

Initial experiments were conducted with five of the labeled AQUA peptides but the ratios of light to heavy peptides exhibited a wide range, which differed by seven-fold among peptides even though samples were spiked with identical amounts of internal standard. Further, large variations were observed for each peptide determination (3–22 %). Therefore, the accuracy and precision of quantification of ApoA-I were highly dependent on which particular peptides were chosen. A similar problem was addressed by the Hoofnagle group [29, 30]. They showed that the coefficients of variation (CVs) of measurements that included the digestion step were more than two-fold higher than the CVs of measurements performed with predigested samples. Additional causes of this inaccuracy may include differences in the behavior of the peptides versus protein from sample extraction step to protein digestion step.

**Fig. 4:**
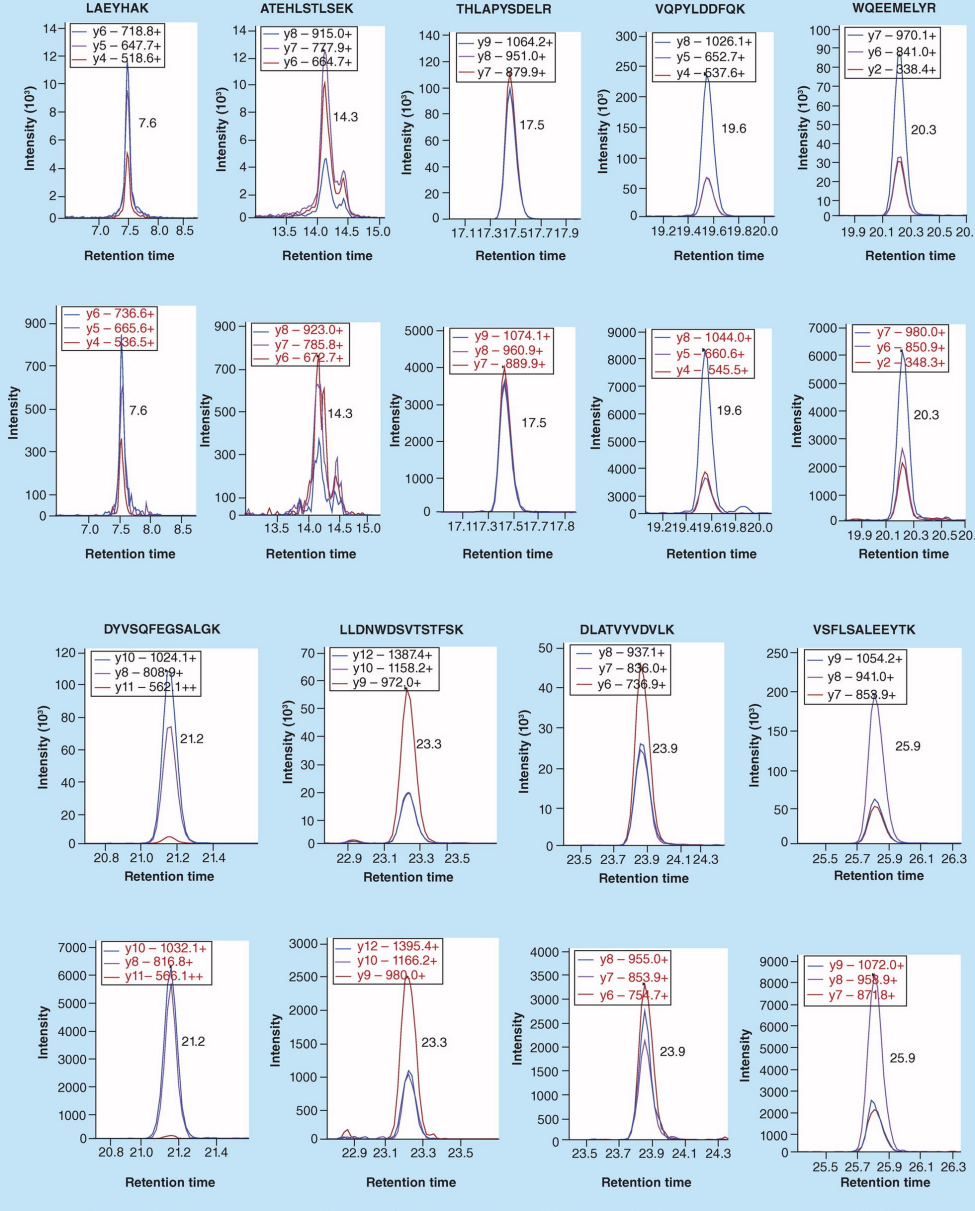
Typical LC–MRM/MS chromatograms of three ion transitions for nine signature ApoA-I peptides (*upper*) and their stable isotopically labeled (heavy) analogs (*lower*). Reprinted with permission from [4]

Initially 15 potentially useful tryptic peptides were selected for potential use in the quantitative analysis of ApoA-I according to the ranking in the Peptide Atlas. These were reduced to nine peptides, which included all eight of the tyrosine-containing tryptic peptides and spanned amino acids D^13^ to K^238^ of the 243 amino acid protein. LC–MRM/MS analysis was conducted using three transitions for each of the peptides and their corresponding stable isotope peptide standard peptides (Fig. 4).

The mean concentrations (±standard deviation) of ApoA-I in nonsmokers and smokers were 169.4 (±55.1) and 138.2 mg/dl (±48.9), respectively (Fig. 3b). The smokers had statistically significant lower serum ApoA-I concentrations compared with nonsmokers (*p* < 0.01). All nine serum ApoA-I tryptic peptides also showed the same trends in both groups, with a statistically significant reduction in eight of the peptides in smokers compared with nonsmokers. Peptides VQPYLDDFQK and VSKLSALEEYTK showed good correlation in both nonsmokers (r^2^ = 0.98) and smokers (r^2^ = 0.90). That confirmed that the spike in SILAC-labeled ApoA-I internal standard procedure corrected for variability in peptide forming during the hydrolysis procedure. Tyrosine-192 has been reported as the predominant site of both nitration and chlorination by myeloperoxidase in vivo and was also the major site of nitration by peroxynitrite (ONOO-) in vitro. In this study, the correlation of peptide LAEY192HAK (r^2^ = 0.88) was good in nonsmokers, while poor (r^2^ = 0.64) in smokers. These data also provide additional confirmation that evaluation of trypsin digestion efficiency before choosing final peptides for quantification as suggested previously by Miller et al. can further improve the precision and accuracy of protein quantification.

Quantification using SILAC labeled proteins has been used in numerous proteomics studies. Rigorous method validation using a full-length stable isotope labeled protein internal standard has been used rarely. The methodology can be employed to explore subtle changes in ApoA-I levels as a potential biomarker of cardiovascular disease and as a biological response indicator for tobacco smoking to complement the use of urinary nicotine metabolites. It is noteworthy that the AQUA internal standards did not provide adequate accuracy and specificity for the analysis of serum ApoA. The stable isotope dilution LC–MS assay for serum ApoA-I is complex and can only be performed in a limited number of laboratories. Unlike widely used simpler and less specific assays such as ELISAs, it provides an accurate and highly reproducible assessment of serum ApoA-I concentrations. Reduced levels of serum ApoA-I are associated with an increased risk for CHD (such as for smokers) and so this assay has significant clinical potential in identifying individuals at risk. The assay will also have clinical utility for providing definitive information on the effects of smoking cessation as well as therapeutic interventions in other high risk populations with HMG-CoA-reductase inhibitors such as rosuvastatin and atorvastatin and fibrates such as fenofibrate or dietary interventions with saturated and cis-monounsaturated fatty acids. Such individualization of therapy is currently not possible with the assay methodology that is available for analyzing serum ApoA-I concentrations in most clinical laboratories.

### ApoC-III

ApoC-III is an 8.8 kDa protein consisting of 79 amino acids and so is much smaller than ApoA-I [31]. It is synthesized in the liver but unlike ApoA-I once secreted it resides on the surface of LDL and very low density lipoprotein (VLDL) as well as HDL [32]. Clinical studies have revealed that increased serum ApoC-III is a potential cardiovascular disease risk factor [33, 34]. The role of ApoC-III in cardiovascular disease is related to its ability to impair plasma lipoprotein metabolism, which leads to increased TAG levels [32]. In addition, ApoC-III has direct atherogenic properties through its ability to stimulate the adhesion of monocytes to endothelial cells and induce the production of inflammatory mediators [35, 36]. This result is consistent with the finding that a genetic defect in the production of ApoC-III is associated with reduced TAGs and reduced atherosclerosis [37]. A systematic review of 5 retrospective and 7 prospective studies provided consistent evidence for an association of cardiovascular events with elevated blood ApoC-III levels in plasma or in VLDL and LDL [32]. However, it was concluded that more data would be required in order to determine the importance of levels of ApoC-III in specific lipoproteins for cardiovascular risk assessment and management [32]. Several hypolipidemic agents including fibrates and statins have been reported to decrease ApoC-III levels [24]. Furthermore, there is a clear association between elevated TAGs and ischemic vascular disease and ischemic heart disease [38]. Consequently, ApoC-III, which is involved in the elevation of serum TAGs, has become a significant therapeutic target [38, 39]. In order to determine the efficacy of new therapeutic agents it will be important to use the most specific and sensitive assay methodology possible.

Most studies of ApoC-III levels in serum, VLDL, and LDL that have been conducted to date employed immunoassay-based methodology [40–43]. However, the relatively small size of ApoC-III and its high abundance in plasma makes it amenable to more specific MS-based methodology. A landmark study conducted by Jian et al. [5] used a “top down” approach coupled with stable isotope labeled protein standards. The method, which employed LC-high-resolution time-of-flight (HR-TOF)/MS made it possible to quantify different glycoisoforms of intact ApoC-II in human plasma.

**Fig. 5:**
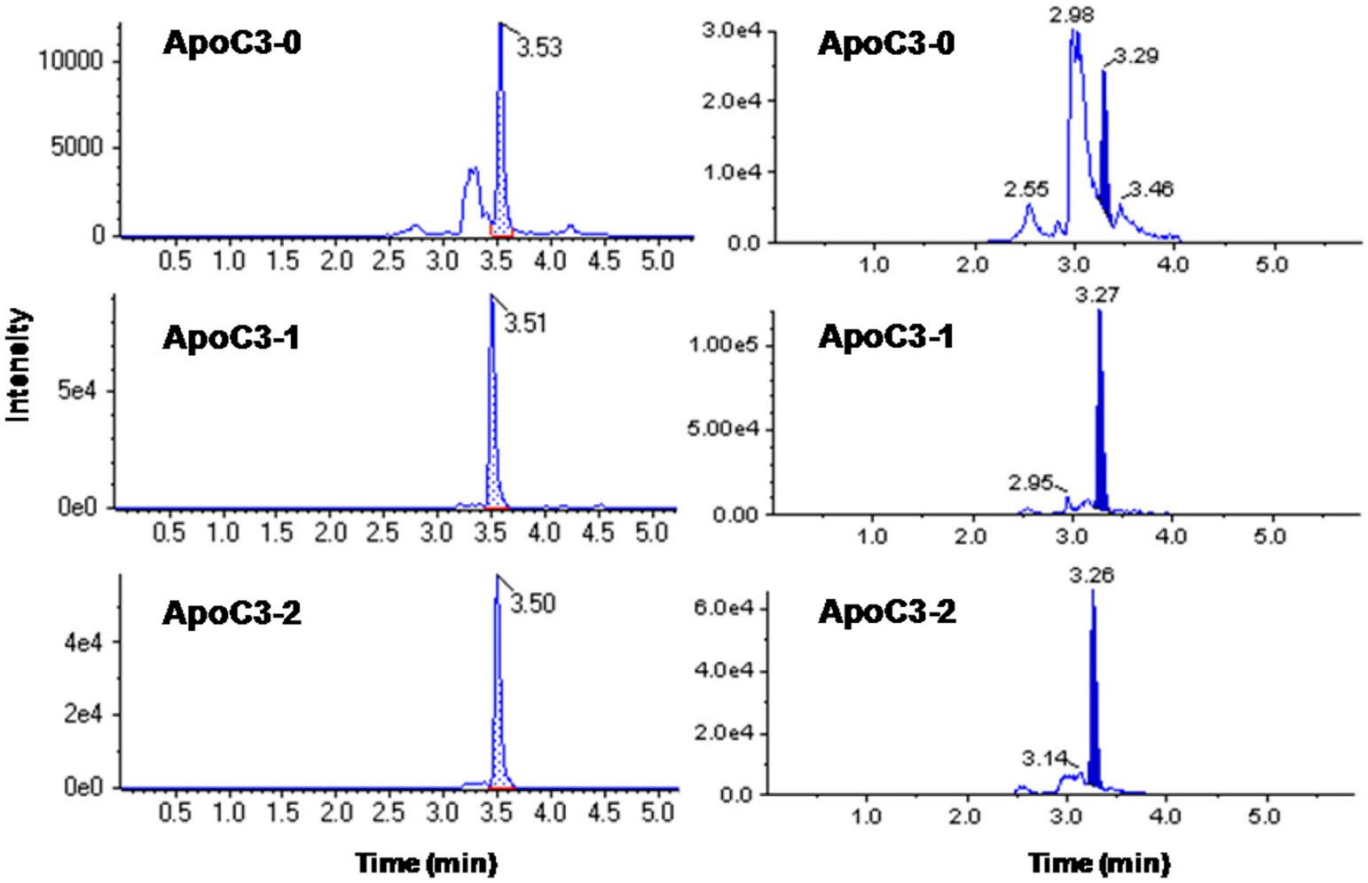
Comparison of chromatography obtained from LC–HR/MS analysis (*left panel*) and LC–MRM/MS analysis (*right panel*) of ApoC-III (ApoC-III-0) and its glycoforms (ApoC-III-1, ApoC-III-2) in human plasma. Reprinted with permission from [5]

Glycosylation enhances the structural stability or function of ApoC-III [34]. Plasma ApoC-III is found primarily in the unmodified form together with two different glycoisoforms [44, 45]. O-linked glycosylation, which occurs on a threonine-74, consists of galactose, N-acetyl-galactosamine, together with one or two N-acetylneuraminic acid (NeuNAc or sialic acid) residues [5]. In order to address this issue a relative quantitation method for glycoisoforms of intact ApoC-III in human plasma using LC–HRMS was developed by Jian et al. [5] and compared with an LC–MRM/MS method (Fig. 5). A fast solid-phase extraction procedure was utilized to clean up the plasma samples, which were then subjected to LC–HR full scan MS analysis. The three most abundant isotopic peaks at charge state 5 and 6 were extracted using a narrow window (50 mDa). The peak area ratio of different glycoisoforms was then calculated and used in an assay evaluation and a biomarker study. It was demonstrated that reproducibility of the peak area ratio was excellent in plasma obtained from multiple subjects and over extended times and various storage conditions.

The LC–HRMS method was applied in a preliminary biomarker research study for sample analysis of plasma obtained from normal, prediabetic, and diabetic subjects. The results showed that there was significant difference in the ratios of ApoC-III-1/ApoC-III-0 and ApoC-III-2/ApoC-III-0 among the different groups. Data from complete full scan HRMS experiments can also be mined for other PTMs and proteins. Utilization of an internal standard also afforded the possibility of evaluating the association of absolute abundance of the glycoisoforms to other diseases. The workflow can be easily extended to other glycoprotein biomarkers of similar sizes. For glycoproteins of larger sizes, immunoaffinity enrichment of the targeted protein and proteolytic digestion may be needed. The relative quantitation approach in these cases is expected to be valid for biomarker research of certain diseases. During the study, the chromatographic spectra showed that glycosylated ApoC-III-0, ApoC-III-1 and ApoC-III-2, were the more abundant species, and they were present at charge states of 5, 6, 7, 8, and 9. ApoC-III-0 gave a much weaker signal, and it could only be observed at charge states 5 and 6. The calculated difference between the observed and theoretical values was less than 10 ppm for all ions, indicating excellent MS accuracy. Near-baseline separation of the isotopic peaks was achieved.

Peak integration for the study was conducted on the three most abundant ions, 1570.9104, 1571.0773, and 1571.2444 (theoretical values), using an extraction window of 50 mDa. The noise between the isotope peaks was excluded, eliminating potential interference. The three most abundant isotopic peaks at charge state 5, 1884.8924, 1885.0927, and 1885.2933 (theoretical values), were also extracted. The same procedure was performed for ApoC-III-1 and ApoC-III-2. Next, the ratio of ApoC-III-1 chromatographic peak area to that of ApoC-III-0 and the ratio of ApoC-III-2 peak area to that of ApoC-III-0 were calculated and further used in assay evaluation and sample analysis. An internal standard was used to asses general quality of the assay. A stable isotope labeled ApoC-III-0, was synthesized by replacing Ala76, Ala78, and Ala79 with [^2^H_4_]-Ala and was spiked at the beginning of the sample preparation procedure. Horse plasma was used as a surrogate matrix. In preliminary experiments, the horse plasma did not contain interference components to human ApoC-III. The peak areas of ApoC-III-0, ApoC-III-1, and ApoC-III-2 were directly used in peak area ratio calculations. The internal standard was used for evaluation of absolute abundance. The ratio of each species to the internal standard observed in each sample can be calculated to compare the absolute abundance of that glycoisoform in different subjects. This technique may be suitable for other protein biomarkers where the absolute rather than the relative abundance is more relevant to the pathological or pharmacological effects to be investigated. The method evaluation was conducted to elucidate the capability of the assay to reproducibly obtain the ratio values upon repeated analysis (intraday and interday), as well as after exposure of the samples to room temperature, freeze–thaw cycles, and long-term storage.

The full scan LC–HRMS-based “top-down” approach affords several major advantages in comparison to “bottom-up”, as well as MRM-based “top-down” approaches. In the “bottom-up” approach, selected peptides generated from enzymatic digestion are monitored as surrogates of the protein. In comparison, the HRMS “top-down” work flow allows acquisition of information from the whole protein, as well as multiple PTM species in a single run. In addition, full scan HRMS is more efficient than the MRM-based. There is no prerequisite to know the expected parent ion and product ion, and a generic MS method can be used. This HRM approach can also provide better specificity than an MRM-based approach. Not having to find a good fragment ion was particularly useful for ApoC proteins as it was found that there were no suitable product ions that could be generated in collision-induced dissociation. As a result, “pseudo-MRM”, i.e., monitoring of parent ion to parent ion with very low collision energy, had to be conducted, which significantly sacrificed the specificity of the assay. In comparison, the specificity of HRMS was independent of fragmentation.

Newer HR instruments can provide enough resolution to separate the isotope peaks of the targeted glycoisoform from the noise, as well as give high mass accuracy. Multiple isotope peaks at each charge state can be extracted using a very narrow window to avoid interference. Usually, the interference peaks are at a much lower relative level in LC–HRMS than in LC–MRM analysis, especially for ApoC-III-0, which was present at the lowest abundance among all three species. Nontargeted approaches are always desired in order to conduct post acquisition data mining to reveal other components, such as biotransformation products, different PTM species, or even other proteins. The data set could also be screened for the truncated form of ApoC-III, which has been associated with elevated carboxypeptidase due to pancreatic disease. If the association of ApoC-III with other diseases is found, then the data set can offer retrospective information. Mining the patient data can be conducted on an as-needed basis to reveal important information relevant to the disease status or treatment outcome, as well as to identify other potential biomarkers.

### High sensitivity (HS) CRP

**Fig. 6:**
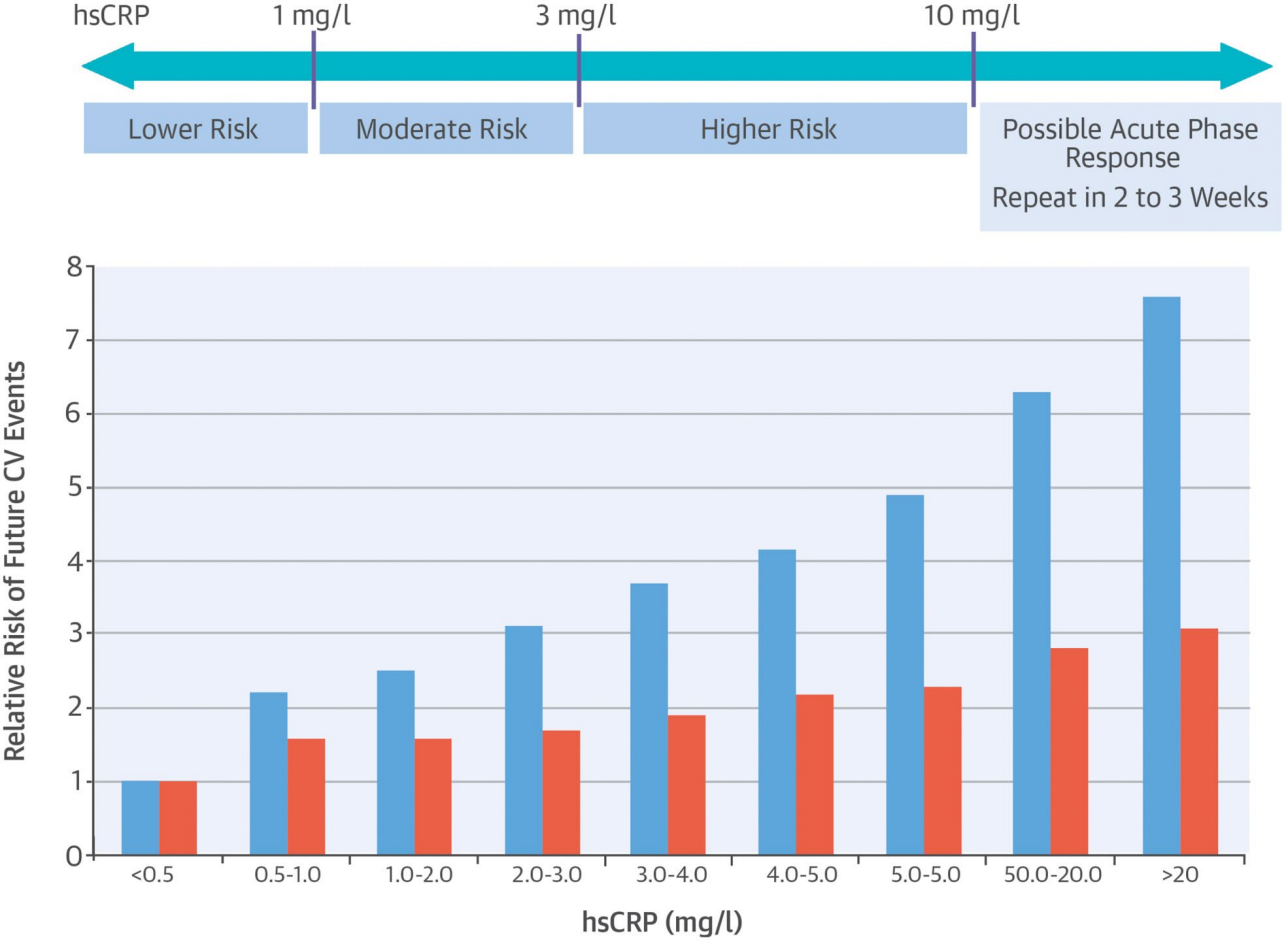
The relationship of inflammation to cardiovascular risk is linear across a wide range of hsCRP values. *Blue bars* represent crude relative risks; *red bars* represent relative risks adjusted for traditional Framingham risk factors. Reprinted with permission from [51]

CRP was originally discovered in serum as an acute phase response in pneumonia infection [46]. It was subsequently shown to be one of some forty plasma proteins that are increased after an inflammatory stimulus [47]. CRP is produced mainly by hepatocytes primarily in response to cytokine interleukin (IL) 6, an activity that is enhanced by IL-1β [48]. CRP is a 45.1 kDa member of the small pentraxins family of proteins comprising 224 amino acids, which exist in an annular pentameric discoid shape [47]. It is an inflammatory mediator that has been widely accepted as an important risk indicator that can independently predict future cardiovascular events and mortality [49–51]. For example, in a meta-analysis of hsCRP levels involving 160,000 subjects each standard deviation in log normalized hsCRP was associated with an increased risk of 1.37 for CHD [52]. Although, there are a number of confusing issues that limit the definitive use of hsCRP as a biomarker of cardiovascular disease [53], some useful guidelines have been developed to address the relationship between hsCRP levels and cardiovascular disease risk (Fig. 6) [51].

Assays for hsCRP are typically conducted using ELISA methodology with a purified protein standard, a monoclonal anti-CRP antibody, and a diluted plasma or serum sample [54–60]. There are numerous suppliers of ELISA kits that can quantify serum or plasma CRP levels down to 1 µg/L. Surprisingly, there have been very few studies to compare ELISA with MS-based methods or to examine whether any post-translational modifications (PTMs) to CRP such as acetylation are associated with cardiovascular disease. There is one study, which described the use of immuno-matrix-assisted laser desorption/ionization (MALDI)/MS for the analysis of serum CRP [61]. No PTMs on CRP were detected by MALDI/MS [61]. None of the four reported LC–MS methods are ideal for protein quantification [62–65]. Three of them rely on the addition of stable isotope labeled peptide internal standards to the protein digestion [62–64]. This allows tracking of any losses during the subsequent LC–MS procedure but does not correct for differences in rates of protein digestion or for differential degradation of the labeled peptides during hydrolysis [4]. It is noteworthy that there were significant differences in the plasma CRP levels between the LC–MS assay and established ELISA reported in one of the LC–MS studies [64]. Unusually, the ELISA method provided significantly lower levels for plasma CRP than the LC–MS method. One study used a standard addition approach using unlabeled CRP protein [65], which does not have the rigor that would have been possible if stable isotope labeled CRP protein had been available at the time of the original study [66]. Therefore, there is a real need for a gold standard method in which stable isotope labeled CRP is used as the internal standard [66] to ensure that there are no confounding variables (such as differential degradation of the peptide standards) during the hydrolysis step of the LC–MS procedure. The availability of this assay could potentially provide data to establish improved specificity for hsCRP as a biomarker of cardiovascular disease.

Williams and Muddiman used a protein cleavage (proteolysis) coupled with isotope dilution (PC-ID) MS methodology for the standardized measurements of CRP. The ideal PC-IDMS requires complete protein cleavage, which produces a 1:1 molar ratio between the initial intact protein and the peptide or peptides to be analyzed. The quantification using IDMS is based on the ratio of the response of the labeled internal standard peptide to that of the unlabeled peptide resulting from the digestion of the particular protein of interest. The ability to quantify proteins with PTMs has been demonstrated by having PTMs incorporated onto synthetic internal standard peptides. CRP was quantified in human plasma without the use of immunoaffinity chromatography or other separation techniques using PC-IDMS. The method used nanoflow LC and a triple quadrupole mass spectrometer operating in SRM/MS mode. The results from the LC–SRM/MS assay were compared with the results from an immunoassay test. The method was used to measure CRP levels in ovarian cancer patients but can be easily used in cardiovascular patients. The reduction in sample processing minimizes the loss of the analyte of interest and minimizes the variance in analytical results caused by the implementation of immunoaffinity and size exclusion separations. The addition of the internal standard to plasma at the beginning of the process accounted for any losses in processing.

Four peptides were synthesized and utilized as possible internal standards based on a tryptic digestion of purified CRP analyzed on a hybrid LTQ-Fourier transform-ion cyclotron resonance mass spectrometer. The synthesized peptides were then dissolved and examined for purity and one naturally occurring peptide ESDTSYVSLK (tpCRP14–23 tryptic peptide from CRP, amino acids 14–23) was used as a standard. The concentrations of both the tpCRP14–23 and the isotopically labeled form used as an internal standard were verified by the Scopes method, which allowed for quantification of the stock solutions. The transitions used for the naturally occurring form and the internal standard minimized the interferences from other possible tryptic peptides that occur from tryptic digestion of human plasma. The method was evaluated for robustness after running more than 100 plasma samples and was found to be very reliable even if a new column needed to be used for the last set of the samples. The use of the internal standard peptide and the specificity of SRM/MS afforded the quantification of the tpCRP14–23 in the presence of other components from the plasma samples.

All samples analyzed by the PC-IDMS method were sent to an independent laboratory to have an ELISA analysis performed for validation. ELISA utilized a polyclonal antibody and was designated as a wide range/high sensitivity CPR test. The linear regression for the comparison of the values obtained by LC–MRM/MS and ELISA had R^2^ = 0.9708 which indicated a good correlation between CRP concentrations determined utilizing the PC-IDMS method and the ELISA wide-range hsCRP test. However, the concentrations obtained by PC-IDMS were much higher. This disparity in the values reported from different detection methods means that a reference range must be determined for each method, such that the values attained can provide appropriate information.

Several years later, Kilpatrick et al. [66] expressed ^15^N-labeled CRP in *Escherichia coli* and *Pichia pastoris* to be used as an internal standard in MS-based assays. Bacterial heterologous protein expression has the benefit of producing large quantities of protein but often has severe limitations regarding protein solubility, folding structure and PTMs-particularly disulfide bonds, which are often crucial to correct protein folding. Yeast expression systems offer the closest environment resembling the human cell for the nascent protein, including homologous chaperone proteins, non-reducing cytoplasm and secretion mechanisms but usually in lower yields. ^15^N-labeled rCRP (^15^N-rCRP) was generated in both bacterial and eukaryotic expression systems for use as the internal standard in LC–MS [66].

The vector pCRPWT5 was sequenced to verify the encoding of the CRP protein (amino acids 19–224) without its native secretory signal sequence. The vector was transformed into the *E. coli* bacterial strain and rCRP protein expression was induced for 24 h using ^15^NH_4_Cl as the sole nitrogen source in the culture medium. Final purification was performed with a p-aminophenylphosphorylcholine column utilizing the binding affinity of the pentameric form of CRP for phosphorylcholine. The production of ^15^N-rCRP was extremely inefficient and the yield was too low to be practical. They also expressed rCRP in *P. pastoris* for the generation of ^15^N-labeled protein. Eukaryotic systems benefit the heterologous expression of CRP by avoiding aggregation and protein mis-folding associated with bacterial systems, presumably due to the conservative nature of the chaperone proteins and similarities in cytoplasmic and organelle composition. Protein expression was induced by methanol addition in minimal medium containing (^15^NH_4_)_2_SO_4_ as the sole nitrogen source leading to the secretion of ^15^N-labeled rCRP into the culture medium. The cells were removed from the medium, which was directly applied to a p-aminophenylphosphorylcholine column and purified in a one-step process. The yield was about 50-fold greater than with *E. coli*. Mass determination of newly purified protein was performed by MALDI–MS that indicated a main peak at *m/z* 23,705 instead of the expected *m/z* 23,307 for ^15^N-rCRP. The mass difference was consistent with the mass of the sequence, EAEA, which is known to be incompletely processed from some proteins in this expression system. MALDI MS/MS analysis of the sequence of this peptide also confirmed the addition of the EAEA sequence on the amino terminus.

The average [^15^N]-incorporation percentage was 98.2 %, making this approach useful for generating internal standard for LC–MS. The ability of the ^15^N-rCRP to produce linear responses within the mass spectrometer was assessed by digesting a series of samples of purified human CRP (ranging from 0.3 to 2 μg) each spiked with 1 μg of ^15^N-rCRP. The ratios of the protein quantities, as well as their response ratios, is a true reflection of the intended use of the labeled protein as an internal standard for which it must have a linear relationship to native CRP in the trypsin proteolysis and subsequent mass spectrometry. The ratio of the mass of the unlabeled to labeled CRP was compared to the ratio of the peak response areas for each corresponding protein. There was a linear relationship of the ratios (r^2^ > 0.99). The slope of the regression line would be expected to be equal to 1 in order to truly indicate that full digestion equivalency was achieved for the ^15^N-rCRP. The slope was slightly less than 1 probably due to the uncertainty associated with the stock concentrations of protein. The difference shouldn’t impact the use of ^15^N-rCRP as an internal standard because of the strong linear relationship, which exists to the native CRP. A common application of a labeled internal standard in ID–MS is to use as a constant volume spike. As long as the concentration of the spiked material is of appropriate scale to the expected value, the analysis will be normalized to the volume of the spiked material and the actual concentration of the spike is not required for concentration assignment as long as the protein concentration of the standard CRP used for the calibration curve is accurate.

LC–MS/MS analysis of increasing quantities of purified human CRP with constant quantity of *P. pastoris* expressed ^15^N-rCRP was done after digestion with trypsin. A total of 12 transitions from 5 peptides were monitored for both the labeled and natural forms. The response ratio was calculated by dividing the response area for the natural form by the corresponding response area of the labeled form. The response ratio of all the peptides were averaged at each amount and plotted against the corresponding mass ratio of natural-to-labeled CRP. Use of the expressed ^15^N-rCRP as an ID–MS internal standard provides the capability to normalize an analysis for a number of aspects such as recovery, digestion, volume transfers, MS ionization and instrument response drift. Accounting for these variables reduces measurement uncertainties in development of the quantification methods required for the generation of needed reference materials in the standardization of this important clinical analyte.

In the report by Kuhn et al. [62], synthetic peptides of CRP containing a uniformly labeled [^13^C]-leucine were added to tryptic digests of human serum and analyzed on a triple quadrupole mass spectrometer by MRM/MS. This provided selective, sensitive, and reproducible detection of both the labeled peptide and the corresponding endogenous CRP peptide. Quantification of CRP levels in patient serum was determined by measuring the ratio of the endogenous tryptic peptide of CRP against the [^13^C]-labeled synthetic peptide standard. For each patient sample analyzed by the method, the values obtained by MS were compared to the results of an immunoassay that is specific for CRP. Synthetic peptides representing the sequences of four trypsin cleavage products of CRP were used: ESDTSYVSLK, QDNEILIFWSK, APLTKPLK, and GYSIFSYATK. A corresponding set of uniformly [^13^C]-labeled leucine-containing peptides was also synthesized for three peptides, ESDTSYVS[^13^C_6_]LK, QDNEI[^13^C_6_]LIFWSK, and AP[^13^C_6_]LTKP[^13^C_6_]LK, while in the case of the fourth peptide, a uniformly [^13^C]-labeled leucine was used in place of the naturally occurring isoleucine residue, GYS[^13^C_6_]LFSYATK (^13^C-CRP peptides). Peptide purity was assessed by reversed-phase chromatography and MALDI-TOF–MS. The sequences of the synthetic peptides were confirmed by MS/MS analysis of the precursor ions on a triple quadrupole mass spectrometer. MS/MS spectra for each peptide were generated on the doubly charged precursor ions and five sequence-specific transition ions were selected for subsequent MRM/MS analyses.

Pooled serum samples (1 mL) were diluted with two volumes of 200 mM ammonium bicarbonate buffer solution, and subjected to affinity depletion of abundant proteins to remove HSA, IgG, and haptoglobin. This process is estimated to enrich the remaining serum components by effectively removing up to 90 % of the abundant proteins, as assessed by SDS-PAGE, thus enabling up to a 10-fold or greater enrichment of the proteins remaining in the mixture. [^13^C]-CRP peptides were spiked into the tryptic digest for use as internal standards in the quantification of the corresponding endogenous peptides derived from the trypsinization of CRP [67]. Their study illustrates that the use of isotope-labeled synthetic peptides and MRM/MS is a powerful analytical method for the prescreening of candidate protein biomarkers in human serum such as CRP prior to antibody and immunoassay development [67].

### HMGB1

**Fig. 7:**
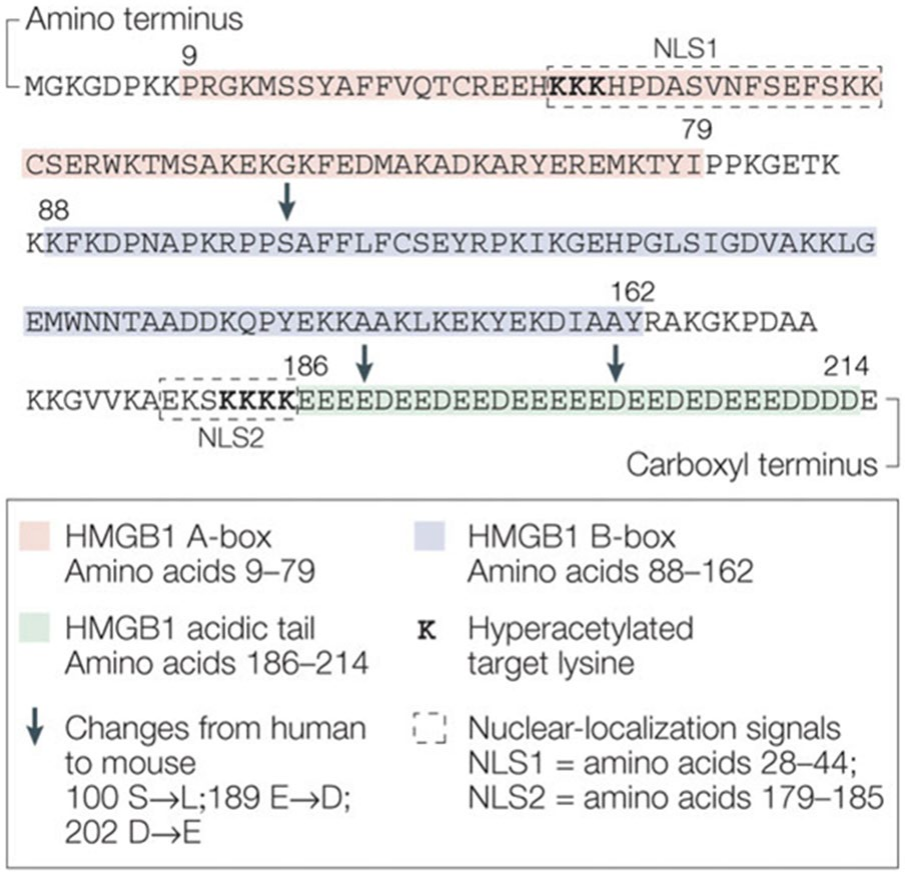
HMGB1 structure. A linear diagram of high-mobility group box 1 protein (HMGB1) is shown, including the residues that constitute the A-box (*pink*), B-box (*purple*) and acidic tail (*green*). The proximal A-box and B-box of HMGB1 both contain putative nuclear-emigration signals, as identified by binding to the nuclear exportin chromosome-region maintenance 1. HMGB1 also contains 43 lysine residues, some of which are frequently acetylated in lipopolysaccharide-activated macrophages (shown in bold). These lysine residues are found within two nuclear-localization signals (indicated by *dashed boxes*): NLS1, which spans amino acids 28–44; and NLS2, which spans amino acids 179–185. Reprinted with permission from [72]

Some 40-years ago, HMGB1 was identified as a non-histone protein that is present in nucleus [6, 68, 69]. It is 30 kDa DNA-binding protein consisting of 215 amino acid residues that are organized into three domains, which include two tandem HMG box domains (A box and B box) that are arranged in an L-shaped configuration, and a C-terminal tail of 30 amino acid residues (Fig. 7) [70, 71]. The nuclear localization of HMGB1 is due to the presence of two lysine-rich nuclear localization sequences (NLSs) spanning amino acids 28–44 (NLS1) and 179–185 (NLS2) [72]. HMGB1 is released from macrophages and monocytes by endogenous pro-inflammatory cytokines such as tumor necrosis factor (TNF), interleukin [IL]-1β, interferon [IFN]-γ [68]. Because its N-terminus lacks a signal sequence, HMGB1 cannot be released via the classical endoplasmic reticulum–Golgi secretory pathway. Instead, activated macrophages/monocytes acetylate the HMGB1-three of lysine residues in the NLS1 and five of the lysine residues in NLS2 (Fig. 7) [72], which leads to translocation into the cytoplasm and release into the extracellular milieu [73, 74].

HMGB1 is a pro-inflammatory cytokine that appears to be involved in atherosclerosis and other cardiovascular diseases [6]. For example, activated vascular smooth muscle cells were shown to be a source of HMGB1 in human advanced atherosclerotic lesions and it appeared to directly stimulate the production of both CRP and matrix metalloproteinase through receptor for advanced glycation end product [75]. These findings suggest that HMGB1 produced by activated vascular smooth muscle cells could contribute to the progression of atherosclerosis by increasing the ability of atherosclerotic lesions to rupture [75]. In addition, experimental models of acute coronary syndromes and cerebrovascular accidents showed that HMGB1 is involved in the amplification of the inflammatory response that occurs during acute ischemic injury as well as in the recovery and remodeling that occurs after ischemia [69]. It is noteworthy that patients with acute coronary syndromes or stroke were found to have significantly higher serum levels of immunoreactive HMGB1 than healthy controls [76]. Furthermore, the increased HMGB1 levels were associated with disease severity and mortality [69]. Finally, diet-induced atherosclerosis in ApoE-deficient mice could be prevented by HMGB1 expression using ethyl pyruvate [77]. In addition to its potential role in inflammation and cardiovascular diseases, HMGB1 appears to be involved in drug-induced liver injury [78], alcoholic liver disease [79], pancreatic cancer [80], renal cancer [81], and mesothelioma [82]. However, in drug induced liver disease, alcoholic liver disease, and mesothelioma, the hyperacetylated form of HMGB1 is more important than the unmodified form. Interestingly, in alcoholic liver disease, serine-34 is also phosphorylated in the hyperacetylated HMGB1 (Fig. 7) [79].

**Fig. 8:**
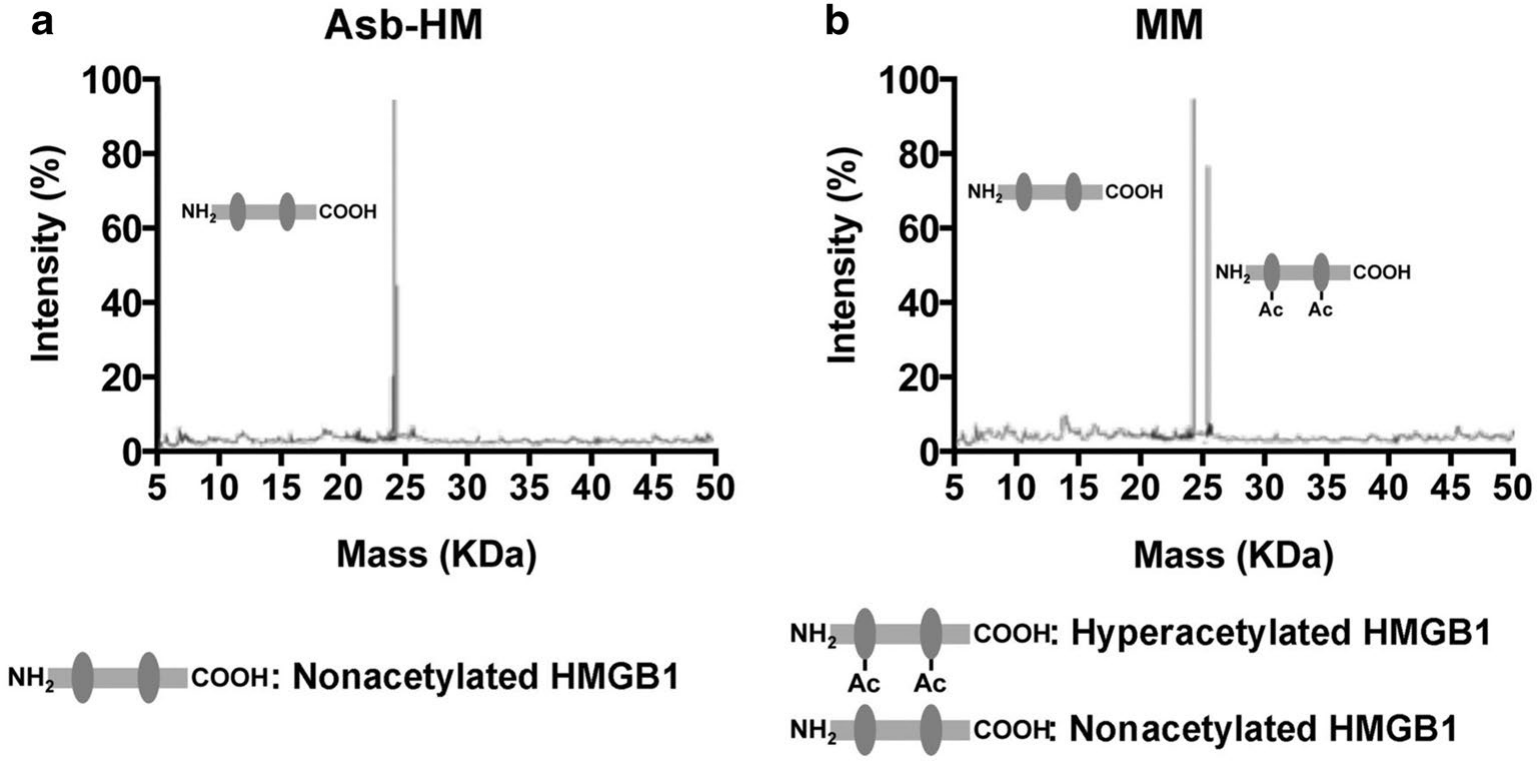
Asbestos-exposed human mesothelin (HM) and malignant mesothelioma (MM) cells release different HMGB1 isoforms. **a** Representative spectrum of whole protein ESI/MS analysis of HMGB1 in crocidolite asbestos-exposed HM where only nonacetylated HMGB1 was detected. **b** Representative spectrum of whole protein ESI/MS spectrum of HMGB1 in malignant mesothelioma cells where both hyperacetylated and nonacetylated HMGB1 were detected. Reprinted with permission from [82]

The quantification of HMGB1 has generally been conducted using immunoassay-based methodology such as ELISAs [83–87]. More recently, high specificity MS-based methods that were developed by Antoine and his colleagues have been employed for HMGB1 analysis [79, 82, 88]. This has made it possible to distinguish the HMGB1 hyperacetylated forms from the unmodified form normally found in the nucleus (Fig. 7) [72]. The MS-based methods are based upon the use of electrospray ionization (ESI)/MS analysis of intact HMGB1 protein followed by spectral deconvolution (Fig. 8) [82] or by the use of Glu-C protease digestion of the HMGB1 followed by ESI/tandem MS (MS/MS) analysis of the hyperacetylated NLS2-derived decapeptide K^180^SKKKKEEEE^189^, which contains five acetylated lysine residues [79].

**Fig. 9:**
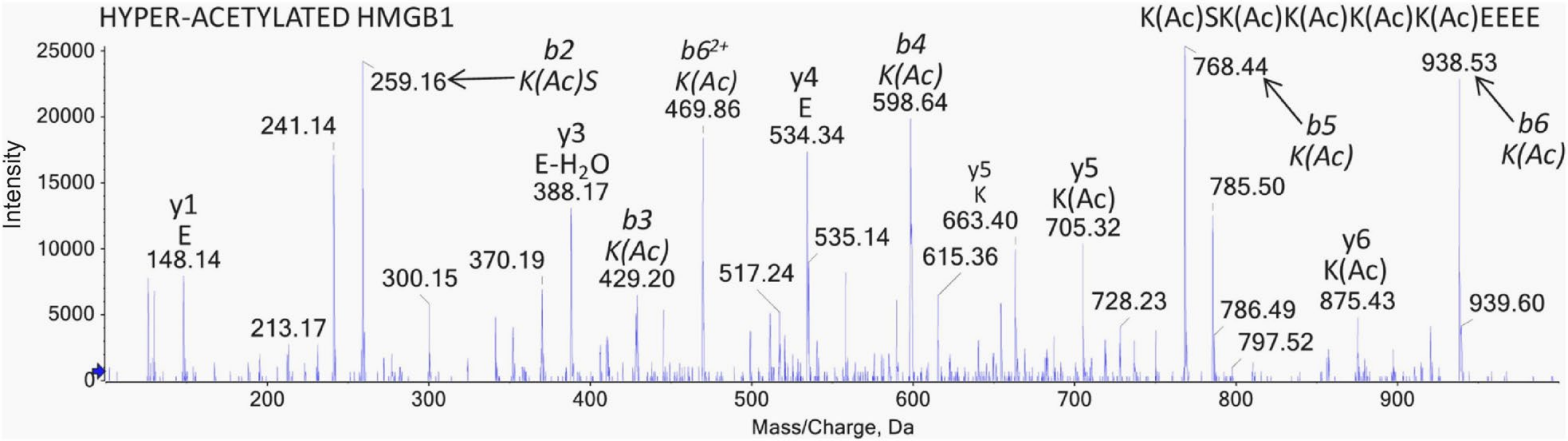
Diagnostic LC–MS/MS spectrum of Glu-C-derived peptide confirming the identification of hyper-acetylated HMGB1 derived from inflammatory cells present in patient sera during acetaminophen hepatotoxicity. Amino acids, b and y ions and peptide sequences are indicated on each spectrum. Acetylated lysine residues within HMGB1 are represented by K(Ac). Reprinted with permission from [88]

Much of the MS-based methodology for analysis of HMGB1 and its acetylated isoforms has emerged from studies of drug induced-liver injury [88]. These methods have not yet been applied to studies of cardiovascular disease but they have already led to the discovery of a highly specific serum biomarker of mesothelioma [82]. LC–MS analysis of synthetic acetylated peptide from the NLS2 region of HMGB1 - K(Ac)SK(Ac)K(Ac)K(Ac)K(Ac)EEEE, revealed that it could be rapidly adsorbed on plastic surfaces [88]. As a result, glass vials were required throughout the assay procedure. To minimize losses during LC–MS analysis a desalted tryptic digest of human serum albumin was used as a proteinaceous carrier for the synthetic peptide. They used a hybrid triple quadrupole-linear ion trap mass spectrometer and injected the synthetic peptide in a background of 2.4 pmol/μL human serum albumin digest. HMGB1 was isolated from 1 mL serum digested with Glu-C. The acetylated peptide was identical with the acetylated authentic standard peptide from NLS2 (Fig. 9). MRM/MS transitions were based on the doubly charged parent ion of *m/z* 736.8 (Fig. 9) and were selected as follows: 736.8/259 (y2–H_2_O), 736.8/341 (internal fragment ion K(Ac)K(Ac)), 736.8/428 (b3) and 736.8/598 (b4); the sodium adducted (doubly charged; *m/z* 747.8) and potassium adducted (doubly charged; *m/z* 755.3) peptides were also included in the method. LC–MRM/MS analysis of serum revealed that acetylated HMGB1 was only elevated in patients that died or required a liver transplant or those that had a worse prognosis. Acetylated HMGB1 therefore holds potential as a prognostic indicator of drug induced liver injury.

In a more recent study by Ge et al., HMGB1 was quantified by LC–ESI/MS in serum and liver from a mouse model of alcoholic liver disease and from the serum of patients with alcoholic liver disease. Acetyl modifications were confirmed on lysine residues (K(Ac)) after LC–MS/MS analysis, for peptides spanning NLS1 (amino acids 27–39) (I) and NLS2 (amino acids 180–188 (179–187 minus methionine) in HMGB1 derived from mice fed ethanol. In addition, a PTM was identified as serine-35 phosphorylation by LC–MS/MS analysis of NLS1 in HMGB1 [79]. This modification was also observed in samples from subjects with alcohol-induced liver injury. Analysis of serum from mice and human subjects with alcoholic liver disease by LC–ESI/MS also revealed an increase in oxidation of HMGB1 to the disulfide isoform. Hepatocytes appeared to be a major source of these different HMGB1 isoforms. This suggests that hepatocyte HMGB1 participates in the pathogenesis of alcoholic liver disease and that HMGB1 undergoes PTMs that are involved in the mechanism of hepatotoxicity [79].

### IGF-I

IGF-I is a 70 amino acid 7.7 kDa protein, which acts as the principal mediator of the effects of growth hormone (GH) [89, 90]. It is largely synthesized in the liver (75 %) and, to a lesser extent, in peripheral tissues [91, 92]. GH directly regulates the production of IGF-1 and its principle transport protein (IGF binding protein 3; IGF-BP3) [93]. Numerous studies have shown that lowered circulating IGF-I levels are associated with an increased risk for cardiovascular disease [94–98]. Measuring serum GH levels have not proven to be a particularly useful diagnostic tool because of the wide range of concentrations that were found [99]. By analyzing the peak GH response to a provocative test with insulin [100], GH secretory status can be assessed for the diagnosis of GH deficiency. Severe GH deficiency is defined by a peak GH response to insulin of >3 µg/mL [101]. Insulin-induced hypoglycemia is unpleasant and contraindicated in patients with seizure disorders or ischemic heart disease [93]. In contrast, serum IGF-I circulates in a much narrower concentration range relative to GH, which means that it can be used as a biomarker for the clinical assessment of diseases related to GH deficiency or excess [102, 103].

IGF-I circulates primarily in a complex bound to its major carrier protein (IGFBP-3) and an acid labile subunit. Consequently, it is necessary to disrupt this complex before analysis, an important factor in the development of assays for serum IGF-I [99]. However, it has been noted that IGF-I immunoassays are prone to interferences from IGFBPs [99] and that there is a lack of standardization and poor inter-assay agreement among the various IGF-I immunoassays [104–107]. A recent report showed that MS-based methodology can be employed in quantitation of serum IGF-I and promises to overcome many of the problems that have been encountered with these immunoassay procedures [99].

Ketha and Singh [99] have developed a LC–HRMS method for serum IGF-I quantitation. Serum was mixed with a buffer containing acidified ethanol to precipitate large proteins and to disrupt the IGF-I/IGFBP complex followed by centrifugation, neutralization, and a cooling step. IGF-I has a relatively small size and remains soluble in the supernatant. An SPE cleanup was employed before injection into LC–HRMA. Oxidized rat IGF-I (ratIGF-IOx) was used as the internal standard. Quantification was performed using standards made from recombinant IGF-I. The LC–HRMS method [108] method was extensively used in over 2000 samples and compared with the iSYS IGF-I automated immunoassay. A reference range was generated and the two methods were greatly correlated, with a reproducible systematic bias. The LC–HRMS method identified 16 outliers where the concentrations were lower than the ones from the iSYS immunoassay. Of the 16 samples examined, 15 had LC–HRMS IGF-I concentrations of approximately 50 % of those obtained by the iSYS assay, whereas 1 sample had a MS result of <5 % of the immunoassay measurement. Reexamination of the HRMS data of these 16 samples led to the discovery of a protein variant with a mass *m/z* 1098 that showed a spectrum with similar isotopic ratio as the monitored *m/z* 1093 charge envelope for IGF-I (most-abundant observed *m/z* 1093.5214, [M + 7H]^7+^). When the protein variant peak was quantitated and summed with the IGF-I results, the LC–HRMS IGF-I values closely matched those of the iSYS immunoassay, leading to the conclusion that the protein variant was related to IGF-I. Additional LC–HRMS IGF-I analysis revealed that this variant was present in approximately 0.6 % of the patient population.

Using the LC–HRMS method, a common variant A70T-IGF1, was identified. The variant could not be distinguished from wild-type by the available immunoassays. When known pathogenic mutations exist or discordant results with established immunoassays are discovered, variant protein sequences should be considered and evaluated. Furthermore, when LC–MS assays give unexpectedly low results, the possibility of the presence of variants should be considered. Consequently, the LC–HRMS method can provide opportunities for more definitive genetic family testing that would be missed by immunoassay procedures [99].

## Summary and future directions

Protein quantification using SILAC labeled proteins has become the method of choice for proteomics studies [109–111]. However, rigorous method validation using full-length correctly folded stable isotope labeled protein internal standards is still under-utilized [13]. We recently developed an assay for serum ApoA-I using LC–MRM/MS together with nine tryptic peptides generated from native ApoA-I and SILAC-labeled ApoA-I [4]. This methodology can now be employed to explore subtle changes in ApoA-I levels as a potential biomarker of cardiovascular disease and as a biological response indicator for tobacco smoking to complement the use of urinary nicotine metabolites [112], NNAL [113], isoprostanes [114], and 8-oxo-dGuo [115]. It will also serve as a gold standard to validate other LC–MS and immunoassay-based procedures for serum ApoA-I. It is also noteworthy that the AQUA internal standards (rather than an intact labeled protein) did not provide adequate accuracy and specificity for the analysis of serum ApoA-I [29, 30].

There are an increasing number of examples where MS-based protein quantification provides important data [3–5, 64, 79, 82, 99, 108] that cannot be obtained with than less labor intensive and cheaper immunoassay-based procedures. It is anticipated that this will lead to significant advances in a number of important issues related to the role of specific proteins in cardiovascular diseases [14, 17, 43, 51, 69, 116]. The availability of a new generation of high resolution high sensitivity mass spectrometers will greatly facilitate these studies as it will be possible to analyze important proteins with levels of specificity and sensitivity that cannot be attained by immunoassay-based procedures. The gain in specificity and sensitivity will more than compensate for the extra complexity and cost. At the very least, MS-based protein quantification methods can provide “gold standards” to confirm that immunoassay-based procedures are accurately quantifying the correct analyte (Table 1).

The stable isotope dilution LC–MRM/MS assays for serum proteins are complex and can only be performed in a limited number of laboratories. However, the assay will have clinical utility for providing definitive information on the effects of therapeutic interventions in high-risk populations. Such individualization of therapy is currently not possible in most clinical laboratories, but with the actual advances of the new instruments it is likely that this technique will start to be used in clinical settings.

## Abbreviations

AQUA: absolute quantification
ApoA-I: apolipoprotein AI
ApoC-III: apolipoprotein CIII
CE: cholesteryl ester
CETP: cholesteryl ester transfer protein
CHD: coronary heart disease
CRP: C-reactive protein
CV: coefficient of variation
ELISA: enzyme-linked immunosorbent assay
ESI: electrospray ionization
HDL: high density lipoprotein
HMGB1: high mobility group box-1
HR: high resolution
hsCRP: high-sensitivity CRP
IGF-I: insulin-like growth factor I
IGFBP: IGF binding protein
IL: interleukin
LCAT: lecithin-cholesterol acyltransferase
LC: liquid chromatography
LDL: low density lipoprotein
MALDI: matrix-assisted laser desorption/ionization
MRM: multiple reaction monitoring
MS: mass spectrometry
MS/MS: tandem MS
NLS: nuclear localization sequence
NSAID: non-steroidal anti-inflammatory drug
PC-ID: protein cleavage coupled with isotope dilution
Platelet Ac-COX-1: acetylated platelet cyclooxygenase-1
PTM: post-translational modification
SILAC: stable isotope labeling by amino acids in cell culture
SRM: selected reaction monitoring
SR-B1: scavenger receptor class B type 1
TAG: triacylglycerol
tNSAID: traditional NSAID
TNF: tumor necrosis factor
TOF: time-of-flight
tp: tryptic peptide
VLDL: very LDL
